# Plant-Growth-Promoting Rhizobacteria Emerging as an Effective Bioinoculant to Improve the Growth, Production, and Stress Tolerance of Vegetable Crops

**DOI:** 10.3390/ijms222212245

**Published:** 2021-11-12

**Authors:** Manoj Kumar, Ved Prakash Giri, Shipra Pandey, Anmol Gupta, Manish Kumar Patel, Atal Bihari Bajpai, Sasha Jenkins, Kadambot H. M. Siddique

**Affiliations:** 1 Institute of Plant Sciences, Agricultural Research Organization, Volcani Center, Rishon LeZion 7505101, Israel; 2 Division of Microbial Technology, CSIR-National Botanical Research Institute, Lucknow 226001, India; vpgiri1989@gmail.com; 3 Department of Chemical Engineering, Indian Institute of Technology, Bombay 400076, India; shipra2589@gmail.com; 4 Department of Biosciences, Faculty of Sciences, Integral University, Lucknow 226026, India; anmolgupta632@gmail.com; 5 Department of Postharvest Science of Fresh Produce, Agricultural Research Organization, Volcani Center, Rishon LeZion 7505101, Israel; patelm1402@gmail.com; 6 Department of Botany, D.B.S. (PG) College, Dehradun 248001, India; dratalbajpai@gmail.com; 7 The UWA Institute of Agriculture and UWA School of Agriculture and Environment, The University of Western Australia, Perth, WA 6001, Australia; sasha.jenkins@uwa.edu.au

**Keywords:** biofertilizer, organic farming, PGPR, vegetables, abiotic stresses, biotic stresses

## Abstract

Vegetable cultivation is a promising economic activity, and vegetable consumption is important for human health due to the high nutritional content of vegetables. Vegetables are rich in vitamins, minerals, dietary fiber, and several phytochemical compounds. However, the production of vegetables is insufficient to meet the demand of the ever-increasing population. Plant-growth-promoting rhizobacteria (PGPR) facilitate the growth and production of vegetable crops by acquiring nutrients, producing phytohormones, and protecting them from various detrimental effects. In this review, we highlight well-developed and cutting-edge findings focusing on the role of a PGPR-based bioinoculant formulation in enhancing vegetable crop production. We also discuss the role of PGPR in promoting vegetable crop growth and resisting the adverse effects arising from various abiotic (drought, salinity, heat, heavy metals) and biotic (fungi, bacteria, nematodes, and insect pests) stresses.

## 1. Introduction

Vegetables are an important component of food and nutrition as they provide energy, vitamins, body-building nutrients, and minerals for human health [1]. Vegetables, fruits, and nuts now play an instrumental role in nutrition, food security, and combating the triple load of malnutrition [2]. The World Health Organization (WHO) proposed the daily consumption of 400 g of edible vegetables and fruits to fulfill the requirements of various micronutrients and prevent noncommunicable diseases [3]. In 2018, the worldwide vegetable seed market was valued at USD 9.163 billion and estimated to increase annually by 9.4% from 2019 to 2024 [4]. Commercially, potato, tomato, cabbage, lettuce, and sweet pepper are important vegetable crops in the global seed market, sharing more than 30% of the total vegetable crop production. However, a wide range of vegetables needs to be consumed to meet dietary requirements.

Potato (*Solanum tuberosum* L.) is a staple, nutrient-intensive, short-duration crop grown in 79% of countries [5]. Tomato (*Solanum lycopersicum*) is widely cultivated worldwide due to its versatility, high dietary fiber and vitamin content, and health benefits. It is a major source of lycopene and antioxidants that can potentially reduce the risk of cancer, osteoporosis, and cardiovascular disease [6]. Cabbage (*Brassica oleraceae*) also provides a range of nutritive and health benefits, including anticarcinogenic, antioxidant, and anti-inflamantory properties [7]. A wide variety of lettuce crops are cultivated across the world, and they are renowned for their high content of phenolic compounds that are beneficial to human health [8]. Pepper (*Capsicum annuum* L.) is widely cultivated in East Asia, including India [9]. It is rich in ascorbic acid, vitamins, and protein and exhibits medicinal properties. Its high ascorbic acid content and its pungent nature make it a popular herbal remedy.

In addition to the major crops, cucumbers, which belong to the Cucurbitaceae family, are important vegetables due to their economic and nutritional value. Immature cucumbers are used for pickles, and the mature fruit are used for salads. The fruit is soft, succulent, and rich in water, vitamins, and potassium (K). In addition to dietary fiber, cucumber contains copper, pantothenic acid, manganese, magnesium, and phosphorus (P) [10]. Cucumber is used in antipyretic and astringent recipes since the fruits and seeds have cooling properties [11]. Broccoli (*Brassica oleracea*) belongs to the Brassicaceae family and is eaten as a vegetable in many countries. It exhibits many health benefits and contains good-quality phytochemicals [12]. Broccoli inflorescences contain hydroxyl cinnamic acids, flavonoids, glucosinolates, and other beneficial compounds with antimicrobial, cardioprotective, anticancer, antioxidant, hepatoprotective, gastroprotective, and anti-inflammatory properties [13]. Several health benefits are associated with broccoli due to its high vitamin (A, B1, B2, B5, B6, C, and E) and mineral (Mg, Ca, Fe, and Zn) contents and the presence of several antioxidants [14]. Among vegetable crops grown in tropical and subtropical areas, okra (*Abelmoschus esculentus* L.) is a popular vegetable rich in vitamins, carbohydrates, minerals, and fats [15].

**Figure 1:**
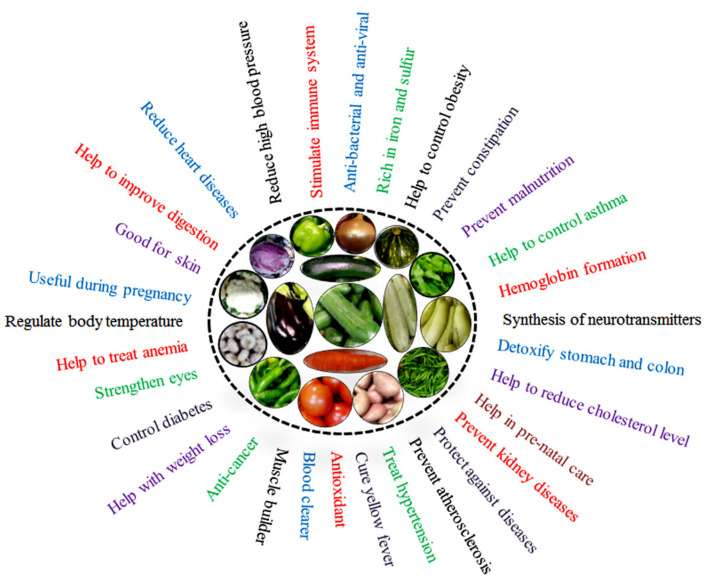
Beneficial effects of vegetables on human health.

Vegetables are important for human nutrition and disease prevention as they boost the intake of calcium, dietary fiber, folate, iron, magnesium, K, and vitamin C [16]. Adequate consumption of vegetables, fruits, and whole grains reduces disease risk and all-cause mortality [17]. Green leafy vegetables have additional human health benefits [18], including a defensive effect against lung cancer [19]. Inadequate vegetable and fruit consumption can lead to chronic diseases, such as blood pressure issues, cardiovascular diseases, osteoporosis, hypercholesterolemia, various types of cancer, respiratory problems, chronic obstructive pulmonary diseases, and mental health issues [20,21,22,23,24] (Figure 1). Increased intake of cruciferous vegetables is associated with a reduced risk of bowel, thyroid, intestinal, lung, and pancreatic cancer [20]. Several varieties of *Capsicum annuum, Lactuca sativa, Allium cepa, Brassica oleracea* var. *sabellica*, and orange-fleshed *Ipomoea batatas* are the richest vegetable sources of phytochemicals with possible anti-obesity activity [25].

Vegetables in the Alliaceae family, including onion, garlic, leek, chive, and Welsh onion, are rich sources of thiosulfides, which are associated with a decline in several chronic diseases [26]. Tomato is the second most consumed vegetable globally after potato, with exclusive nutritional and phytochemical properties. Tomato contains key phytochemicals carotenoids: lycopene 60–64%, phytoene 10–12%, neurosporene 7–9%, and carotenes 10–15% [27]. Parsley (*Petroselinum crispum*) and celery (*Apium graveolens*) are popular vegetables and the best sources of flavonoid apigenin and vitamin E [28]. Carrot (*Daucus carota*) contains a unique combination of three flavonoids—quercetin, kaempferol, and luteolin [29,30,31]—that helps regulate cellular activity and reduce free radicals that cause oxidative stress.

Modern vegetable cultivation depends mainly on chemical fertilizers and pesticides. Chemical fertilizer application is one of the most endorsed systems in developing rigorous agriculture [32,33], leading to increased soil fertility and crop yields. However, the continuous use of chemical fertilizers can result in soil degradation, decreased soil organic matter content and soil quality, nutrient loss via runoff, leaching, and greenhouse gas emissions, leading to air and water pollution [34], pest resistance, and reduced food safety [35].

Organic farming can supply quality food without adversely affecting soil health or the environment. Organic fertilizer improves soil dynamics and increases the soil’s potential to retain water and nutrients in comparison to the effect of chemical fertilizers. Several studies have established that organic farming, which stringently restricts synthetic fertilizer use, is a potential substitute for minimizing the negative effect of chemical fertilizers, with the added benefit that organic farming products usually have enhanced nutritional and soil-quality properties [36,37,38,39]. However, organic farming is associated with lower crop production and higher end-product costs than conventional agriculture. Therefore, chemical fertilizers remain necessary until organic farming significantly increases food production [32,40]. Tomato (*Solanum lycopersicum*), a popular vegetable cultivated in more than 140 countries [41], contains several metabolites that are beneficial for health and nutrition [42]. Organically grown tomato had higher polyphenol, vitamin C, and carotenoid contents than those from conventional farming [36]. Ye et al. [43] reported that bio-organic farming, with decreased rates of chemical fertilization and enhanced soil fertility, produced higher tomato yields and quality than conventional farming. They suggested that *Trichoderma* spp. application as bio-organic fertilizer could be combined with chemical fertilizer application to achieve optimal yields and quality [43]. Thus, an alternative and more sustainable approach amends crops with rhizospheric microbial inoculants (bioinoculants) that promote plant growth and health.

Plant-growth-promoting rhizobacteria are free-living soil microorganisms that naturally colonize the rhizospheric zone of plant roots. These bacteria increase plant growth and control several diseases [44], and they belong to a broad taxonomic diversity, particularly Actinobacteria, Bacteroidetes, Firmicutes, and Proteobacteria. Several bacteria, including *Azospirillum brasilense, Azotobacter salinestris, Burkholderia phytofirmans, Bacillus megaterium, Bacillus subtilis, Paenibacillus favisporus, Paenibacillus polymyxa, Pseudomonas fluorescens, Pseudomonas stutzeri*, and *Rahnella aquatilis*, are consistently part of the PGPR-diversified taxa [45]. These bacteria provide a plethora of plant benefits including increased root growth, nutrient uptake, and plant hormone stimulation, suppression of pathogenic activity, and restoration of soil health through the mineralization of organic pollutants [46,47]. They are not host specific, meaning that they have the advantage of being able to promote the growth of a broad range of hosts. Various rhizospheric bacteria such as *Azospirillum, Azotobacter, Arthrobacter, Alcaligenes, Bacillus, Burkholderia, Enterobacter, Klebsiella, Pseudomonas*, and *Serratia* have been linked with solanaceous vegetable crops [48]. Thus, PGPR are emerging as organic fertilizers suitable for many plant species, which could reduce chemical fertilizer application while enhancing soil quality and plant yield [49]. The PGPR species *Pseudomonas putida* and *Bacillus amyloliquefaciens* decreased the negative impact of three pesticides (carbendazim, imidacloprid, and glyphosate), maintained soil enzyme activities, and enhanced soil health and fertility [50].

Biofertilizer contains living microbes that colonize and promote plant growth by enhancing nutrient availability to the host plant [51]. The application of microbial biofertilizers to seeds or soils promotes the growth and yields of vegetable crops, such as bottlegourd [52], brinjal [53], broccoli [54], cabbage [55], carrot [56], chili [57], cucumber [58], lettuce [59], potato [60], onion [61], pumpkin [62], radish [63], and tomato [64]. The application of *Bacillus* strains improved growth under greenhouse/field conditions of several vegetable crops, such as broccoli, cucumber, lettuce, pepper, and tomato [65,66,67]. The positive role of PGPR on vegetable growth and production is well established [65], involving diverse mechanisms that differ according to the species of bacteria [68], such as the modulation of volatile compound production and hormone content, improvement of nutrient accessibility, and the increase of abiotic stress tolerance [69].

This review summarizes the most updated findings on the role of PGPR as biofertilizers for vegetable crop growth and production. We also discuss the impact of PGPR on vegetables under biotic and abiotic stresses and provide a mechanistic overview for ameliorating several stresses.

## 2. Effect of PGPR in Plant Growth Promotion

**Figure 2:**
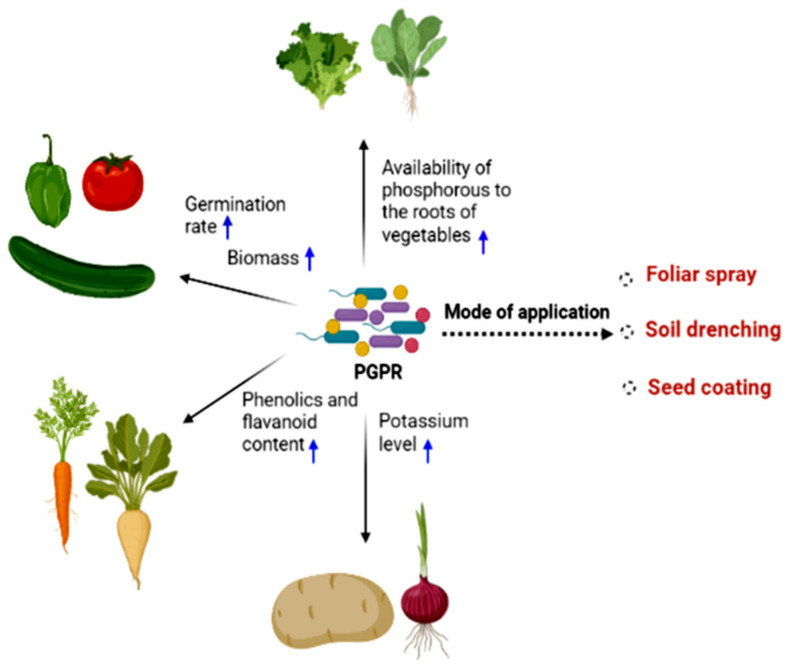
Application of PGPR on vegetables and their anticipated strategies for plant growth promotion. Figure created with BioRender.com (accessed on 2 October 2021).

PGPR play an important role in enhancing soil quality, bioremediation, and stress control to develop eco-friendly sustainable agriculture [67]. PGPR can be used as biofertilizers and biopesticides, improving plant growth through direct mechanisms, such as nitrogen (N) fixation, phytohormone production, and phosphate solubilization (Figure 2). Figure 2 shows the application modes of PGPR bioformulations to plants. Seed coating and soil drenching are the most conventional methods of bioinoculation adopted to promote vegetable growth, whereas foliar sprays are feasible for disease protection. Phosphate-solubilizing bacteria (PSB) are PGPR that hydrolyze organic and inorganic insoluble P compounds into soluble P forms that plants readily use. Bioinoculation with PGPR can increase the germination rate and biomass content and provide essential nutrients (e.g., N, P, K) to plant roots. They also help produce hormones, such as auxin and gibberellins, siderophores, ammonia, and 1-aminocyclopropane-1-carboxylate (ACC) deaminase. Initially, it was assumed that hydrogen cyanide (HCN) production played an important role in plant growth promotion by reducing plant pathogens [70]. Later, the hypothesis changed, and it is believed that HCN production indirectly increases phosphorus accessibility by metal chelation and sequestration and indirectly induces nutrient accessibility to the rhizobacteria and host plants [71]. HCN production by PGPR is independent on genus; thus, they can be used as biofertilizers or biocontrol to increase crop production and yields [72]. The enzyme 1-aminocyclopropane1-carboxylate (ACC) deaminase cleaves the plant ethylene precursor, ACC, into ammonia and ketobutyrate [73]. Decreased ACC levels in plants by ACC deaminase-producing organisms decreased plant ethylene levels [74]; ethylene in high concentrations can lead to plant growth inhibition or even death. PGPR can also increase enzymatic activity and enhance mineral and water uptake [63]. PGPR can protect plants from biotic and abiotic stresses by using indirect mechanisms such as suppressing the growth of plant pathogens and inducing systemic resistance [75,76].

## 3. Role of PGPR in Vegetable Crop Production

Various PGPR can be used as biofertilizers in vegetable crop production. Table 1 provides a list of common PGPR used as biofertilizers on vegetable crops and their application method (seed coating, soil treatment, soil drenching, or foliar spray). Phosphorus is a major nutrient for vegetable growth; in particular, potato (*Solanum tuberosum*) requires high soil P for high biomass production. Limited P supply in soils reduces potato production by about 40% worldwide [77]. Potato needs higher N and P compared to other vegetables due to its tuber formation. Phosphate-solubilizing bacteria enhanced potato tuber growth and biomass production [78]. The synergy between three PSB isolates, *Pantoea agglomerans* strain P5, *Microbacterium laevaniformans* strain P7, and *Pseudomonas putida*, significantly impacted P solubilization and potato production [79]. Moreover, K-solubilizing bacteria can also enhance potato productivity by increasing K availability in the rhizosphere [80].

Cauliflower is an important crop due to its high dietary fiber and nutritional value and belongs to the Brassicaceae family. Cauliflower also benefits from bioinoculation with PSB and other PGPR. Kushwaha et al. [81] reported that the application of PGPR isolates enhanced cauliflower germination and growth by increasing indole acetic acid (IAA) production and P solubilization. Broccoli, known as ‘the crown jewel of nutrition’ due to its high nutritional value, is in high demand worldwide. Broccoli production in India increased after farmers became aware of its high nutritional value and improved cultivation methods. While organic farming could increase broccoli yields by improving nutrient availability to roots [54], Altuntas [82] found that the application of PGPR biofertilizers increased the yield up to 50% and 20% compared to the control and chemical fertilizers, respectively. Broccoli production relies on P absorption from the soil. *Pseudomonas fluorescens*, a solubilizing bacteria, increased broccoli growth when applied with a significant amount of fertilizer [83].

PGPR applied to vegetable crops can act as a biocontrol agent by protecting the plant from pathogens and pests. They achieve this directly by suppressing a broad spectrum of viral, bacterial, fungal, and nematode diseases and indirectly by altering the rhizosphere to favor beneficial microorganisms. Soilborne fungal pathogens that affect vegetable crops, such as *Fusarium* infection in tomato causing wilt disease, are a serious concern worldwide. Nabi et al. [84] evaluated the efficacy of the PGPR *Bacillus aryabhattai* to control *Fusarium* wilt disease in tomatoes and found higher amounts of amino acid and phytohormones in PGPR-treated plants. In addition to *Fusarium*, approximately 80% of tomato crop losses involve *Alternaria solani*, a causative agent of early blight disease [85]. The synergistic effect of green waste and wood biochar mixed with PGPR (*Bacillus subtilis*) inhibited the mycelial growth of *A. solani* by up to 55% in tomato [86]. Tariq et al. [87] evaluated the effect of PGPR on bell pepper (*Capsicum annuum*) yield by applying a consortium of *Klebsiella*, *Burkholderia*, *Panibacillus*, and *Bacillus* spp. in the field for up to 30 days. The results revealed steady yield increases per acre with increasing PGPR formulations. Significant phenotypic and genotypic correlations also occurred between yield per acre and yield in each treatment.

Bioinoculation of PGPR on vegetable crops can support plant growth by alleviating the impact of soil constraints (salinity, acidity, drought). Eggplant (*Solanum melongena*), a member of the Solanaceae family, is cultivated in tropical, subtropical, and Mediterranean countries. Increased Na^+^ uptake in saline soils hampers eggplant growth and yield [88]. However, eggplant seeds treated with PGPR such as *Xanthobacter autotrophicus* BM13, *Enterobacter aerogenes* BM10, and *Bacillus brevis* FK2 decreased Na^+^ uptake and increased K^+^ uptake, which enhanced plant growth [88]. Lettuce (*Lactuca sativa* L.) is sensitive to abiotic stress [89]; its shallow root system makes it sensitive to water deficit, which increases with plant growth [90]. Julia et al. [91] applied a biofertilizer of *Macrocystis pyrifera* algal extracts and the PGPR *Azospirillum brasilense*, which increased germination rate and lettuce growth in saline conditions. In another study, PGPR-inoculated lettuce had a higher phenolic and flavonoid content than uninoculated plants under greenhouse conditions [92]. *Bacillus* and *Pseudomonas* spp. increase salt tolerance in lettuce [67,89]. Okra (*Abelmoschus esculentus* L. Moench), a vitamin- and mineral-rich vegetable widely used by humans, is a secret weapon for diabetic people [93]. *Pseudomonas* spp. colonizes the rhizospheric region of okra roots and enhances plant growth [94].

**Table 1:** Plant-growth-promoting rhizobacteria (PGPR) used as biofertilizers in vegetable production.

PGPR	Vegetable Crop	Mode of Treatment	Effect on Crops	References
*Alcaligenes faecalis* and *Bacillus amyloliquefaciens*	*Spinacia oleracea*	Soil treatment	Mitigated lead toxicity	[95]
*B. pumilus* SE34	*Solanum lycopersicum*	Seed treatment	Induced systemic response during infection	[96]
*Jeotgalicoccus huakuii* NBRI 13E	*S. lycopersicum*, *Abelmoschus esculentus*, *Zea mays*	Seed treatment and foliar spray	Increased yield and ameliorated salt stress	[97]
*B. pumilus* strain SE34 or *B. amyloliquefaciens* strain IN937a or *B. subtilus* strain IN937	*S. lycopersicum*	Seed treatment and soil drenching	Induced resistance against CMV virus	[98]
*Rhizobium* spp.	*S. lycopersicum*, *Capsicum annuum*, *Daucus carota*, *Lactuca sativa*	Seed treatment	Increased biomass	[99,100]
*Bacillus megaterium* var. *phosphaticum*	*S. oleracea*	Soil and seed treatment	Ensured efficient absorption of P, water, and other microelements to alleviate water stress and resist fungal diseases	[101,102]
*Bacillus amyloliquefaciens*	*L. esculentum*	Spraying on leaves	Induced systemic resistance against tomato leaf curl virus disease	[103]
*Bacillus cereus*	*S. lycopersicum*	Soil drenching	Biotic stress resistance against bacterial speck disease caused by *Pseudomonas syringae* pv	[104]
*Paenibacillus alvei* and *Bacillus velezensis*	*Sorghum bicolor*	Seed treatment	Resistance to water stress and crown rot disease caused by *Fusarium pseudograminearum*	[105]
*Pseudomonas fluorescens*	*Arachis hypogea*	Seed treatment	Produced 1-aminocyclopropane-1-carboxylic acid (ACC) deaminase to confer resilience against salinity stress	[106]
PGPR *Bacillus subtilis* (RS_2_) and *Bacillus* spp. (RS_7_)	*C. annuum*	Seedling treatment	Increased productivity	[107]
*Bacillus tequilensis*	*S. lycopersicum*	Seedling and soil drenching	Produced ACC deaminase to confer resilience against salinity stress	[108]
*Stenotrophomonas maltophilia*, *Achromobacter xylosoxidans*, *Achromobacter* spp.	*S. tuberosum*	Potato tuber coating	Increased P solubilization, indole acetic acid, hydrogencyanide, and ammonia	[109]
*Pseudomonas* spp. PS1	*Vigna radiate*	Seed treatment	Increased plant biomass, yield, and protein content	[110]
*B. amyloliquefaciens*	*S. lycopersicum*	Seed treatment	Resistance from bacterial wilt of tomato (*Ralstonia solanacearum*)	[111]
*Bacillus cereus* BC1AW and *Pseudomonas putida* PP3WT	*S. lycopersicum*	Seedling treatment	Ameliorated bacterial wilt disease	[112]
*Pseudomonas fluorescens*	*Solanum tuberosum*	Soil treatment	Protection from *Ralstonia solanacearum* pathogen. Reduced bacterial wilt incidence and improved growth	[113]
*Trichoderma viride* ES1 and *Pseudomonas fluorescens* Bak150	*S. tuberosum*	Foliar spray	Suppressed early blight disease and increased yield	[114]
*Trichoderma* spp.	*Brassica oleracea*	-		[115]
*Trichoderma* spp.	*S. lycopersicum*	Seed priming and soil treatment	Protection from *F. oxysporum* f. sp. *lycopersici*	[116]
*T. harzianum+Pseudomonas* spp.	*S. lycopersicum*	-	Protection from *Sclerotium rolfsii*	[117]
*T. viride+T. harzianum+P. fluorescens+Azotobacter* spp. *+* *Azospirillum* spp. + PSB	*S. lycopersicum*	Seed treatment and soil drenching	Disease management and protection from *Pythium aphanidermatum*, *Ralstonia solanacearum*, *Fusarium oxysporum* f. sp. *lycopersici*	[118]
*Bacillus subtilis*, *Trichoderma* spp.	*S. lycopersicum*, *S. melongena*	Seed treatment	Protection from *Fusarium* infection through secretion of extracellular cell-wall-degrading enzymes	[119,120]
*Pseudomonas fluorescens*	*A. sesculentus*	Seed and soil treatment	Protection from *Rhizoctonia solani* by the producing siderophores, HCN, and indole acetic acid	[121]
*Lactic acid bacteria*	*C. annuum*	Soil drenching and foliar spray	Protection from black rot by producing siderophores	[122]
*Azospirillum brasilense*, *Pseudomonas fluorescens* and *Bacillus megaterium*	*Cucumis sativus*	Seedling treatment and foliar spray	Improved fruit quality	[123]
*Pseudomonas fluorescens*, *Pseudomonas* spp., *Bacillus subtilis*	*C. sativus*	Seed treatment	Protection from damping off by producing antibiotics and metabolites and inducing systemic resistance	[124]
*Chaetomium globosum*, *Burkholderia cepacia*	*S. tuberosum*, *C. annuum*	Soil drenching and foliar spray	Protection from late blight disease by producing endo- and exo-glucanases; antimicrobial activity of organic acids	[125,126]
*Trichoderma harzianum* *+* *Pseudomonas fluorescens*	*S. tuberosum*	Seed treatment and foliar spray	Protection from early blight caused by *Alternaria solani* but active biomolecules not yet determined	[127]
*Bacillus subtilis*	*C. sativus*	Soilless potting mix drenching	Disease suppression against anthracnose disease	[128]
*Stenotrophomonas maltophilia* and *Agrobacterium fabrum*	*Momordica charantia*	Seed coating	Immobilized Cd in Cd-rich soil to improve growth	[95]
*Bacillus velezensis* isolates (Y6 and F7)	*S. lycopersicum*	Soil and seed treatment	Protection from fungal infections by producing antibiotic compounds	[129]

CMV, Cucumber mosaic cucumovirus; P, Phosphorous; HCN, Hydrogen cyanide; Cd, Cadmium.

## 4. Mechanistic Overview of PGPR-Mediated Plant Growth Promotion of Vegetable Crops under Stress Conditions

Plant–microbe PGPR interactions can be divided into two categories—symbiotic bacteria and free-living rhizobacteria, which can be further divided based on indirect or direct actions. Direct mechanisms involve biofertilization, root growth stimulation, rhizoremediation, and biotic and abiotic stress control and indirect mechanisms include disease suppression and induction of systemic resistance [130]. PGPR can be differentiated into two categories depending on their colonization: extracellular PGPR (ePGPR, which inhabit the root surface area) and intracellular PGPR (iPGPR, which colonize the intracellular space of the root cortex) [49]. Several symbiotic bacteria reside in the intercellular spaces of plant cells. Certain bacteria form mutualistic interactions with their host and enter plant cells [131]. Others have physiological interactions with plants and help in structural modifications. For example, rhizobia are well known for their mutualistic behavior, establishing symbiotic interactions with leguminous crops, forming specific root structures (nodules) to fix atmospheric N [132].

Global climate change and land degradation are increasing plant stress due to abiotic factors such as drought, salinity, cold, and heat and biotic stressors such as pathogens and herbivores [133]. PGPR can ameliorate plants from stress conditions [108,134] that affect plant growth through hormonal and nutritional imbalances and physiological and metabolic changes [135]. In addition, PGPR can initiate hydrolytic enzyme production, exopolysccharide production, heavy metal bioremediation, and induced systemic resistance (ISR) stimulation [136]. They also stimulate ISR by accelerating the physical and biochemical responses of plant cells to environmental stresses. PGPR associations with host plants enhance the biosynthesis of defense-related molecules by increasing the level of defense-responsive proteins, which provide survival support under stress conditions. Changes in biochemical and physiological parameters can account for PGPR’s ability to induce stress tolerance through osmolyte production [137], antioxidant production [138], ACC deaminase activity [76], phytohormonal content [139], and biofilm formation [140].

**Figure 3:**
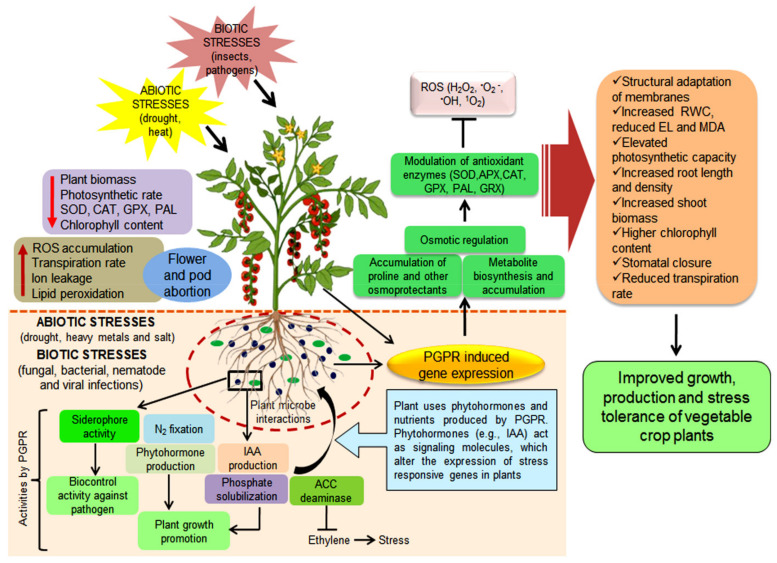
Schematic representation of plant-growth-promoting rhizobacteria (PGPR)-mediated growth promotion and stress tolerance in vegetable crops. The model shows stress-induced reductions in plant biomass; photosynthetic rate; SOD, CAT, GPX, and PAL activities; and chlorophyll content and increases in reactive oxygen species (ROS), flower and pod abortion, transpiration rate, ion leakage, and lipid peroxidation. Plants inoculated with PGPR experience growth-promoting attributes, such as phytohormone (IAA) production and nitrogen fixation, prevent pathogen infections through biocontrol activity, and improve stress tolerance through ACC deaminase activity. PGPR also induce stress-responsive gene expression, leading to the accumulation of several osmoprotectants and defensive compounds and detoxification of ROS in cells. Modulation of antioxidants prevents cell damage and maintains homeostasis. Cellular responses, such as increased relative water content and photosynthetic capacity and reduced ion leakage and transpiration rates, and morphological changes, such as increased root and shoot biomass and reduced flower and pod abortion, occur, which improves growth, yield, and stress tolerance in vegetable crops. IAA, indole-3-acetic acid; SOD, superoxide dismutase; CAT, catalase; GPX, guaiacol peroxidase; PAL, phenylalanine ammonia-lyase. Figure created with BioRender.com (https://app.biorender.com/biorender-templates (accessed on 10 October 2021).

PGPR help plants to resist several abiotic stresses, including drought, salt, cold, and heavy metal toxicity (Figure 3), by colonizing the rhizosphere/endorhizosphere region and producing phytohormones, exopolysaccharides, volatile compounds, and ACC deaminase, which trigger osmolyte and antioxidant production and stress-responsive gene regulation. Salinity affects germination, plant phase transition, plant vigor, and production. Salinity-resistant PGPR induce osmotolerance in plants by improving root and shoot growth, nutrient uptake, chlorophyll content, vigor, and yield. PGPR secrete acids, phytoantibiotics, proteins, and other chemical compounds that help ameliorate toxic heavy metal stress and induce resistance in plants [135].

### 4.1. Role of PGPR against Biotic Stresses in Vegetable Crops

#### 4.1.1. Role of PGPR in Fungal- and Bacterial-Induced Stress in Vegetable Crops

Pathogenic disease control can be triggered by the secretion of extracellular enzymes and other molecules that hydrolyze the microbial cell wall, compete for nutrients in the rhizosphere, and generate ISR against pathogenic infection in plants (Figure 1). For example, *Bacillus xiamenensis* strain PM14 has broad antifungal activity against *Colletotrichum falcatum*, *Fusarium moniliforme*, *Fusarium oxysporum*, *Pythium splendens*, *Rhizoctonia solani*, and *Macrophomina phaseolina*. PGPR produce diffusible and volatile antimicrobial compounds that exert fungicidal effects on phytopathogenic fungi by inhibiting growth or inducing the lysis of fungal mycelia [141]. In plants, PGPR can produce antibiotics (e.g., iturin, surfactins, fengycin, 2,4-diacetylphloroglucinol (DAPG), phenazine), cell-wall-degrading enzymes (protease, chitinase, and cellulase), plant-growth-promoting enzymes, hormones (indole-3-acetic acid), N-acyl-homoserine lactones, and siderophores to suppress pathogen growth [142] (Table 2).

Plant-growth-promoting rhizobacteria can be used as biocontrol agents against phytopathogens. They establish disease resistance in plants by suppressing the pathogens directly or stimulating host plant defenses and competing for nutrients with plant pathogens. Biotic and abiotic stresses confer several physiological changes in plant cells, indicated by the generation of reactive oxygen species (ROS). The accumulation of high ROS levels in plant cells is evident as oxidative damage, disrupting cellular homeostasis. Plant cells are furnished with sophisticated antioxidative mechanisms involving antioxidative defense enzymes, such as ascorbate peroxidase (APX), catalase (CAT), peroxidase (PO), superoxide dismutase (SOD), glutathione reductase, glutathione S-transferase, and guaiacol peroxidase (GPX). These defense enzymes are involved in scavenging and transforming ROS into nontoxic end-products and protecting cells from oxidative damage. In addition, plant cells induce several antioxidant molecules, such as carotenoids and phenylpropanoids, to conquer oxidative damage. Induced systemic resistance primes host plants to resist pathogen colonization through defense-related antioxidative enzymes and molecule production [136]. Other mechanisms, including the production of cell-wall-degrading enzymes, such as β-1-3-glucanase, chitinase, and β-xylosidase; volatile organic compounds; and diffusible antibiotics play key roles during biotic stresses [141].

#### 4.1.2. PGPR against Nematode and Insect Pests

The increasing demand for agriproducts can be met by enhancing yield efficiency and minimizing losses due to plant parasites (nematodes). However, the current chemical-based strategy exerts inappropriate and adverse effects on flora and fauna. There is a need for a biocontrol agent for nematode management, such as PGPR, that can suppress nematodes directly by producing enzymes, toxins, and other metabolic products or indirectly by regulating nematode behavior and altering root diffusates. PGPR induce the production of repellents by the host plant that adversely affect host recognition and alter nematode feeding site development or sex ratios inside root tissue [131]. PGPR also enhance antioxidant activities and improve nutrient uptake by modulating plant hormone levels, increasing root proliferation. *Pseudomonas aeruginosa* enhances proline accumulation and modulates superoxide dismutase activity in tomato infected with *Spodoptera litura*, increasing root and shoot biomass [143].

### 4.2. Role of PGPR against Abiotic Stress in Vegetable Crops

In plants, physiological and chemical changes induced by PGPR that enhance environmental stress tolerance, including that to drought, salinity, cold, high temperature, and heavy metals, are recognized as induced systemic tolerance (IST) [144] (Table 2). These environmental stresses negatively impact endurance, biomass production, and staple food crop yields by up to 70%, affecting food security globally. Aridity stress due to drought, salinity, and high temperature is the leading abiotic stress restricting plant growth and productivity [130]. The application of PGPR against abiotic stresses has been widely studied [63,145,146,147].

**Table 2:** Plant-growth-promoting rhizobacteria (PGPR) mediated biotic and abiotic stress tolerance in vegetable crops.

Stress	Crops	PGPR Isolates	PGP Activity	References
**Abiotic stress**				
Salinity	*Abelmoschus esculentus*	*Enterobacter* spp.	Increased ACC deaminase activity	[148]
Salinity	*Lycopersicum esculentum*	*Streptomyces* spp. strain PGPA39	Increased ACC deaminase activity, phosphate solubilization, and IAA production	[149]
Drought	*L. esculentum*	*Bacillus subtilis*	Cytokinin signaling	[150]
Drought	*Capsicum annuum*	*Bacillus licheniformis* K11	Reduced ethylene concentration	[151]
Salinity and drought	*Cucumis sativus*	*Burkholderia cepacia*, *Promicromonospora* spp.	Increased salicylic acid and gibberellic acid	[152]
Salinity	*Solanum melongena*	*Pseudomonas* spp.	Produced antioxidant enzymes	[153]
Salinity	*Pisum sativum*	*Bacillus* spp.	Increased IAA production, phosphate solubilization, ammonia production, ACC deaminase activity, siderophore production, and antioxidant enzyme production	[154]
Salinity	*Mentha* spp.	*Halomonas desiderata* STR8 and *Exiguobacterium oxidotolerans* STR36	Reduced harmful effects of salinity	[155,156]
Salinity	*M. polymorpha*, *Medicago lupulina*, *Medicago truncatula*, *Medicago sativa*	*Bacillus megaterium* NMp082	Induced tolerance to salt stress	[157]
Heat	*Solanum lycopersicum*	*Bacillus cereus*	Extended thermotolerance in tomato seedlings	[158]
**Biotic stress**				
Damping off	*L. esculentum*	*Streptomyces* isolate DBTB 13, *Trichoderma viride*, *T. harzianum,* and *P. fluorescens + Azotobacter* and *Azospirillum*	Reduced stunting and stem collapse in infected plants	[118,159,160]
Bottom rot	*Lactuca sativa*	*Bacillus amyloliquefaciens* strain FZB42	Improved the quality of lettuce by preventing wilting and rotting	[161]
Powdery mildew	*C. sativus*	*Ampelomyces quisqualis* Ces., *B. subtilis* strain GB03	Prevented crop from tiny white superficial spots, reduced severity of angular leaf spot disease (foliar disease)	[162]
White rust disease, *Fusarium* wilts	*Spinacia oleracea*	*B. subtilis*, *Pseudomonas* spp., *Bacillus* spp., *Burkholderia* spp., *Penicillium oxalicum*, *Enterobacter cloacae*, *Trichoderma* spp.	Controlled *Fusarium* wilt and white rust	[78,163]
*Colletotrichum lindemuthianum*	*Phaseolus vulgaris*	*P. fluorescens*	Disease management against biotic stress	[164]
Damping-off	*Beta vulgaris*	*Pseudomonas fluorescens*	Disease management by producing antifungal compounds	[165]
*Plasmodiophora brassicae*	*Brassicae oleraceae*	*Trichoderma* spp.	Prevented and managed club root disease in cabbage	[115]
*Pythium aphanidermatum*, *Ralstonia solanacearum*, *Fusarium oxysporum* f. sp. *lycopersici*	*L. esculentum*	*T. viride+T. harzianum+P. fluorescens+Azotobacter+Azospirillum +* PSB	Disease management from several biotic stress	[118]
Powdery mildew, *Botrytis* rot	Greenhouse crops	*Ampelomyces quisqualis*, *Pseudomonas flocculosa*, *Ulocladium* spp.	Disease control against *Botrytis* rot and powdery mildew	[166]
Fusarium wilt, bacterial wilt	*S. melongena* and *L. esculentum*	*Trichoderma* spp., *Bacillus subtilis*, *Bacillus amyloliquefaciens*, *Pseudomonas fluorescens*	Produced antibiotics and secondary metabolites to control bacterial wilt and fusarium diseases through the secretion of enzymes that degrade extracellular wall components	[119,120,167]
Root rot disease	*Abelmoschus esculentus*	*Pseudomonas fluorescens*	Disease management by producing siderophores, HCN, and indole acetic acid	[121]
Damping off, downy mildew	*Cucumis sativus*	*Pseudomonas* spp., *Bacillus subtilis*, consortium of *Achromobacter* spp., *Streptomyces* spp., *Bacillus licheniformis*	Disease management by producing numerous antibiotics, metabolites, and induced systemic resistance	[124]
Bacterial spot and blight disease	*C. annuum*	Lactic acid bacteria, *P. fluorescens*	Protection by producing siderophores, numerous chemicals, and microbial fungicides	[122,168]
Late blight	*S. tuberosum*	*Burkholderia cepacia; Chaetomium globosum*	Protection by generating antimicrobial activity through organic acids and enzymes, such as exo- and endo-glucanases	[125,126]
*Pythium aphanidermatum*	*L. esculentum* Mill.	*Streptomyces* isolate H2	Prevented damping off, thus acting as a biocontrol agent	[160]
Squash mosaic virus	*C. sativus*	*P.fluorescens*, *B. polymyxa*	Protection from pathogenic viruses	[169]
Watermelon mosaic potyvirus	*C. maxima*	*B. subtilis*, *B. pumilus*	Biocontrol mechanism for pathogenic viruses	[170]
Bacterial wilt, *Fusarium* wilt, leaf spot, anthracnose, *Alternaria* leaf blight, downy and powdery mildew	*Citrullus lanatus* (Thunb.)	*P. polymyxa* (SN-22), *Sinomonas atrocyanea* (NSB27)	Reduced angular leaf spot lesions and gummy stem blight lesions and inhibited bacterial fruit blotch	[156]
*Fusarium* wilt	*Raphanus sativus*	*Pseudomonas putida* strains WCS358 and RE8	Provided biocontrol mechanism against biotic agent	[156]

ACC, 1-aminocyclopropane-1-carboxylate; IAA, Indole acetic acid; HCN, Hydrogen cyanide.

#### 4.2.1. PGPR-Mediated Drought Tolerance in Vegetable Crops

PGPR such as *Achromobacter*, *Bacillus*, *Citrobacter*, *Mesorhizobium*, *Pseudomonas*, and *Variovorax* could be used to enhance tolerance against drought stress in potato and tomato [171,172]. Tomato needs substantial irrigation water for successful growth, with drought stress significantly decreasing yields [173]. Drought affects potato growth and productivity by changing plant water relations, enhancing oxidative stress, decreasing photosynthetic capacity, inhibiting enzyme activities, and destroying membranes [174]. Drought affects the start of tuberization and decreases the rate of budding and weight of tubers [175]. Drought stress in plants is exacerbated in semi-arid areas in developing countries, leading to significant harvest losses [176]. Several PGPR, such as *Pseudomonas putida*, *Bacillus amyloliquefaciens*, *Azospirillum brasilense*, and *Bacillus subtilis*, play an important role in plants for drought tolerance [177,178,179]. For example, the application of *Bacillus subtilis* HAS31 reduced the impact of drought and maintained potato production (growth rate, dry matter production, leaf area, number of tubers, tuber weight, and yield) under severe water stress [180] by altering plant growth regulators and activities of superoxide dismutase (SOD), peroxidase (POD), and hydrogen peroxidase (CAT). Application of *Bacillus cereus* AR156 to tomato plants also maintained productivity. The mechanisms involved in drought tolerance were attributed to increased SOD, POD, and CAT synthesis and upregulation of cytosolic ascorbate peroxidase gene (cAPX) and monodehydroascorbate reductase gene (MDHAR) [181]. In another study, *Bacillus licheniformis* K11 reduced drought stress in pepper plants by increasing auxin and ACC deaminase production [151].

#### 4.2.2. PGPR-Mediated Salinity Tolerance in Vegetable Crops

Most vegetable crops are affected by salinity stress [182], reducing crop growth and production through changes in morphological and physiological parameters [183]. Salinity stress affects vegetable crop growth due to osmotic or water-deficit stress, salt accumulation in shoots, nutrient imbalance, or a combination of these [184,185]. The ability of PGPR to decrease salinity stress has been evaluated for various vegetable crops [186]. PGPR enhanced salt stress tolerance in okra (*Abelmoschus esculentus*) through ROS-scavenging enzymes and improved water use efficiency [148]. Lettuce is one of the most consumed leafy vegetables and is a comparatively salt-sensitive crop [182,187]. Moncada et al. [67] studied the role of PGPR in enhancing the salinity stress tolerance of leaf lettuce developed in autumn and spring in a floating system by adding a PGPR-based biostimulant containing *Bacillus* spp. to mineral nutrient solutions (MNS) [67], which significantly alleviated salt stress and thus increased plant biomass and improved physiological and morphological parameters. In addition, Saravanakumar et al. [106] studied the effect of PGPR on groundnut in saline-affected soils. PGPR showed ACC-deaminase activity to combat salt stress by modulating antioxidant enzymatic activities. Application of PGPR confers tolerance against salinity stress in several other vegetable crops, including tomatoes, cucumbers [188], eggplant [189], tobacco, mustard, bell peppers, and radish [54].

#### 4.2.3. PGPR-Mediated Tolerance to Heat, Metal Toxicity, and Other Stresses in Vegetable Crops

Elevated temperatures constrain vital plant functions and reduce yield in various agroclimatic zones. It is a major environmental concern globally. However, PGPR have been implicated in heat stress tolerance in several plants (see list in Table 2 and mechanistic overview in Figure 3). Bensalim et al. [190] reported that potato plants inoculated with *Burkholderia phytofirmans* strain PsJN had enhanced survival under high heat stress. Martin and Stutz [191] studied the role of arbuscular mycorrhizal fungi isolates that improved the growth and productivity of pepper (*Capsicum annuum* L.), increasing the amount of dry substance and P uptake at higher temperatures. Similarly, Mukhtar et al. [192] evaluated the efficacy of rhizobacteria *Bacillus cereus* for mitigating the heat stress effect in tomato and found that ACC-deaminase, exopolysaccharides, and the extracellular enzymatic attributes of PGPR modulated tomato growth traits under elevated temperature.

Heavy metals are a major environmental stress with several adverse effects on agricultural production and human health. Heavy metal accumulation in plants leads to their accumulation in the food chain and creates major health issues [193]. Plants require some metals for growth and development, but not all metals are useful. Extreme quantities of metals can act as toxicants that hamper plant growth and production [194]. The application of PGPR-based bioinoculants reduced the negative effect of metals such as copper (Cu), zinc (Zn), cadmium (Cd), nickel (Ni), and lead (Pb) in beans [195], potatoes [196], peas [197], tomato, canola, and Indian mustard [198]. Singh et al. [199] demonstrated the beneficial association of PGPR for alleviating the adverse effects of heavy metals in different crops and vegetables.

## 5. Conclusions and Future Perspectives

Chemical fertilizers can have detrimental effects on the soil, environment, and human health, while biofertilizers are naturally occurring products that do not negatively impact the soil ecosystem or human health. Therefore, PGPR-based biofertilizers are an indispensable and key component of sustainable agriculture to maintain long-term soil fertility and retain crop productivity. PGPR are an emerging biofertilizer alternative for chemical fertilizers to improve agricultural crop production, particularly vegetable production. PGPR promote the growth and production of vegetable crops through a variety of mechanisms, including the provision of phytohormones (e.g., IAA) and improved nutrient absorption (e.g., N, P, K). Considering the positive impact of PGPR as biofertilizer in terms of crop yield and productivity. In addition, PGPR protect plants from various abiotic and biotic stresses through osmotic adjustment, biocontrol activity, siderophore production, and ACC-deaminase production, among others. PGPR are useful soil bacteria that can stimulate biological, chemical, and physical modifications and alleviate the detrimental effects of abiotic and biotic stresses in vegetable crops. Frequent application of PGPR-mediated bioinoculants will enhance vegetable yields and production, particularly under stress conditions. Governments and private agencies should promote biofertilizer use as an environmentally friendly replacement for chemical fertilizers. In addition, farmers need to be educated on the beneficial effects of PGPR-based biofertilizers for sustainable agriculture.

## Data Availability

Data presented in this study are available in the article.

## Abbreviations

AcdS	1-Aminocyclopropane-1-carboxylate deaminase
CAT	Catalase
GPX	Guaiacol peroxidase
IAA	Indole-3-acetic acid
IST	Induced systemic tolerance
PAL	Phenylalanine ammonia-lyase
PGPR	Plant-growth-promoting rhizobacteria
P	Phosphorus
K	Potassium
ROS	Reactive oxygen species
SOD	Superoxide dismutase
WHO	World Health Organization
